# Good’s syndrome and infections in a large retrospective cohort of thymoma

**DOI:** 10.3389/fimmu.2026.1733466

**Published:** 2026-05-28

**Authors:** Wenji Jia, Lei Jin, Xiao Huan, Chong Yan, Jianying Xi, Liewen Pang, Chongbo Zhao, Jie Song, Xianglin Chu, Sushan Luo

**Affiliations:** 1 Department of Neurology, No.2 Affiliated Hospital, Kunming Medical University, Kunming, China; 2 Huashan Rare Disease Center and Department of Neurology, Huashan Hospital, Shanghai Medical College, National Center for Neurological Disorders, Fudan University, Shanghai, China; 3 Department of Thoracic Surgery, Huashan Hospital, Fudan University, Shanghai, China

**Keywords:** Good’s syndrome (GS), immunodeficiency, infection, immunology, overall survival, thymoma auto-immunity

## Abstract

**Introduction:**

Good’s syndrome (GS) is an immunodeficiency associated with thymoma characterized by hypogammaglobulinemia. The co-existence of recurrent infections may be associated with quality of life and overall survival. Despite its relevance, the impact of GS on patients with thymoma has rarely been studied in large cohorts.

**Methods:**

This is a retrospective cohort study of patients with pathologically confirmed thymoma recruited between May 2010 and November 2024. Data on peripheral immune profile, infections, and paraneoplastic syndrome (PNS) were collected and analyzed.

**Results:**

Of 1001 patients with thymoma, we ultimately included 158 patients who underwent peripheral immune profiling. The prevalence of GS was 45.6% (72/158), and the infections were reported in 43.69% (69/158). Infection rates were higher in the GS group than in non-GS group (50% versus 32.72%, *P* = 0.0384). Among the 121 pathogens identified from this cohort, bacteria were the most prevalent (52.07%, 63/121), followed by fungi (34.71%, 42/121) and virus (13.22%, 16/121). Patients with infections had significantly lower overall survival compared with those without infections (*P* < 0.001). In addition, patients with multiple PNS had poorer survival than those with a single PNS (*P* < 0.001). No significant difference in overall survival was found between the GS and the non-GS groups.

**Conclusion:**

GS and infections are prevalent in patients with thymoma. Infections and multiple PNS were associated with poorer survival outcomes. These findings highlight the importance of early detection and intervention for improving prognosis and long-term survival in patients with thymoma.

## Introduction

Thymic epithelial tumors (TETs) encompass thymoma, thymic carcinoma, and thymic neuroendocrine tumors (NETs), all of which originate in the anterior mediastinum (1). Although TETs represent the most common primary tumors in this anatomical compartment, their overall incidence remains low. Thymomas exhibit diverse biological behavior and typically follow an indolent clinical course. Histologically, thymomas are classified into type A (including an atypical variant), type AB, type B (subdivided into B1, B2, and B3), micronodular thymoma with lymphoid stroma, and metaplastic thymoma; immunohistochemical assessment of immature T cells may also aid in classification (2). In contrast, thymic carcinoma generally displays more aggressive behavior and is associated with a poorer prognosis.

As the central organ for T-cell maturation and immune tolerance, the thymus critically shapes the immune system (3). Neoplastic transformation of thymic epithelial cells disrupts thymic architecture and immune homeostasis, resulting in a paradoxical phenotype characterized by both immune deficiency and hyperactivity (4). Despite the well-established link between thymoma and autoimmune diseases, its prognostic significance remains unclear. Data from large databases (e.g., ITMIG, ChART) show that patients with autoimmune diseases often have more favorable clinical features (younger age, female predominance, early stage, benign histology) and may achieve better overall survival. By contrast, certain non-myasthenic autoimmune diseases, particularly pure red cell aplasia, are associated with a poorer prognosis (5, 6). Consequently, patients with thymoma are predisposed to Good’s syndrome (GS) as well as a broad spectrum of paraneoplastic syndromes (PNS) (7).

Although surgical resection offers favorable outcomes for localized thymoma (8), long-term survival may be influenced by thymoma recurrence, immune-related complications, comorbid conditions, and the peripheral immune landscape in patients with thymoma remains unexplored. Emerging evidence suggests that immune dysregulation may be associated with the prognosis of thymoma (9).

GS represents a distinct and severe form of thymoma-associated immunodeficiency. Patients with GS typically exhibit hypogammaglobulinemia, peripheral B-cell depletion, and varying degrees of cellular immune dysfunction, and are at high risk for recurrent or opportunistic infections (10). Despite its clinical significance, current knowledge of GS is largely derived from small case series. Further, comprehensive analyses integrating peripheral immune profiles, infections, GS, and PNS in relation to long-term survival are scarce.

This large retrospective cohort study aims to analyze the prevalence of GS and infections in patients with thymoma. By characterizing clinical profiles and identifying associated risk factors for long-term survival, we aim to improve the monitoring strategies and facilitate the timely initiation of targeted interventions, thereby potentially enhancing survival and quality of life for patients with thymoma.

## Methods

This retrospective study enrolled patients with pathologically confirmed thymoma treated at Huashan Hospital, Fudan University, from May 2010 to November 2024. Inclusion criteria were (1): Pathologically diagnosed thymoma (2); Masaoka-Koga stage I-IV (3); Availability of the consecutive clinical and follow-up data. Patients with thymic hyperplasia, thymic cysts, or thymic carcinoma were excluded.

Clinical variables were collected retrospectively, including sex, age at diagnosis, interval from diagnosis of thymoma to the development of GS, PNS types, overall survival, comorbidities, WHO histological classification, Masaoka-Koga stage, R0 resection status, intravenous immunoglobulin (IVIg), creatine kinase, serum IgG levels, flowcytometry analysis of peripheral T or B lymphocytes, routine laboratory tests, rheumatic antibodies, and detailed infection characteristics (pathogens and infection sites). The WHO and Masaoka-Koga classifications followed guidelines (11). Surgical treatment was classified as thymoma resection, total thymectomy, or extended thymectomy.

Pathogen identification was performed in the context of clinically suspected infections. Since infectious episodes occurred during myasthenic crisis requiring invasive mechanical ventilation or intensive care, microbiological evaluation was primarily based on conventional bacterial and fungal cultures, including sputum, bronchoalveolar lavage fluid, blood, and urine samples. In cases with suspected invasive fungal infection, fungal PCR assays were additionally performed according to clinical indications. Mixed infections were defined as the identification of two or more distinct pathogens from a single patient, either during infectious episode or from different anatomical sites. To minimize the inclusion of contamination, only pathogens detected in a compatible clinical context and confirmed by repeated positive cultures or PCR results were analyzed.

GS was primarily defined as the coexistence of thymoma and hypogammaglobulinemia (IgG <8.7g/L). In patients who had received IVIg before immunological evaluation, GS was additionally supported by markedly reduced peripheral B-cell counts on flow cytometry. This approach acknowledges that exogenous immunoglobulin supplementation can normalize serum IgG levels, thereby masking an underlying humoral immunodeficiency. Myasthenia gravis (MG) is characterized by fluctuating muscle weakness, in combination with positive anti-acetylcholine receptor (AChR) antibodies and/or abnormal repetitive nerve stimuli (RNS). Myocarditis required exclusion of other cardiac diseases.

Statistical analyses were performed using GraphPad 10.4. Continuous variables expressed as mean ± SD, and categorical variables as frequency (%). Group comparisons employed Mann-Whitney U or t-tests for continuous variables, and Fisher’s exact test or chi-square tests for categorical variables, as appropriate. Overall survival and cumulative event risk were analyzed with Kaplan-Meier method and the log-rank test.

## Results

Among 1001 patients with pathologically confirmed thymoma, 158 patients (15.78%) with serum IgG and peripheral lymphocytes measurements were included in the final analysis. The mean age at evaluations was 52.22 ± 13.62 years, with the mean age at the diagnosis of thymoma being 47.34 ± 14.72 years. The cohort included 72 males and 86 females, with an average follow-up duration of 61.82 ± 63.91 months. The overall mortality in this cohort was 11.39% (18/158).

According to the Masaoka–Koga staging, this cohort was comprised of 24.68% (39/158) with stage I, 36.71% (58/158) with stage II, 9.49% (15/158) with stage III, 29.11% (46/158) with stage IV. Based on the WHO histological classification, 15.19% (24/158) were type AB, 15.82% (25/158) type B1, 46.84% (74/158) type B2, and 22.15% (35/158) type B3 thymoma. The proportion for R0 resection was 51.90% (82/158).

Among all participants (n=158), the mean absolute cell count for peripheral CD4^+^ T cells was 511.8 ± 376.7 (389.09-1046.98/ul) (n=132), with the proportion of 34.72 ± 12.16% (21.69-49.76%) (n=151). The mean absolute CD8^+^ T cell count was 476.3 ± 360.1 (219.42-987.26/ul), with a proportion of 34.36 ± 11.71% (12.84-44.61%) (n=132). Peripheral CD20^+^ B counts were 165.6 ± 155.8/ul (94.54-374.47/ul) (n=129), accounting for 11.39 ± 8.47% (6.35-18.97%) (n=148). A reduction in either the absolute cell counts or proportion was observed in 31.01% (49/158) of the patients for B cells, and in 87.97% (139/158) for CD4^+^ T cells. Hypogammaglobulinemia was identified in 57 patients (57/158), fulfilling the primary diagnostic criterion for GS. Additionally, among the remaining 101 patients with normal IgG levels, 15 patients (14.85%) who received IVIg maintenance therapy exhibited reduced peripheral B lymphocytes counts or proportions and were classified as GS.

**Figure 1:**
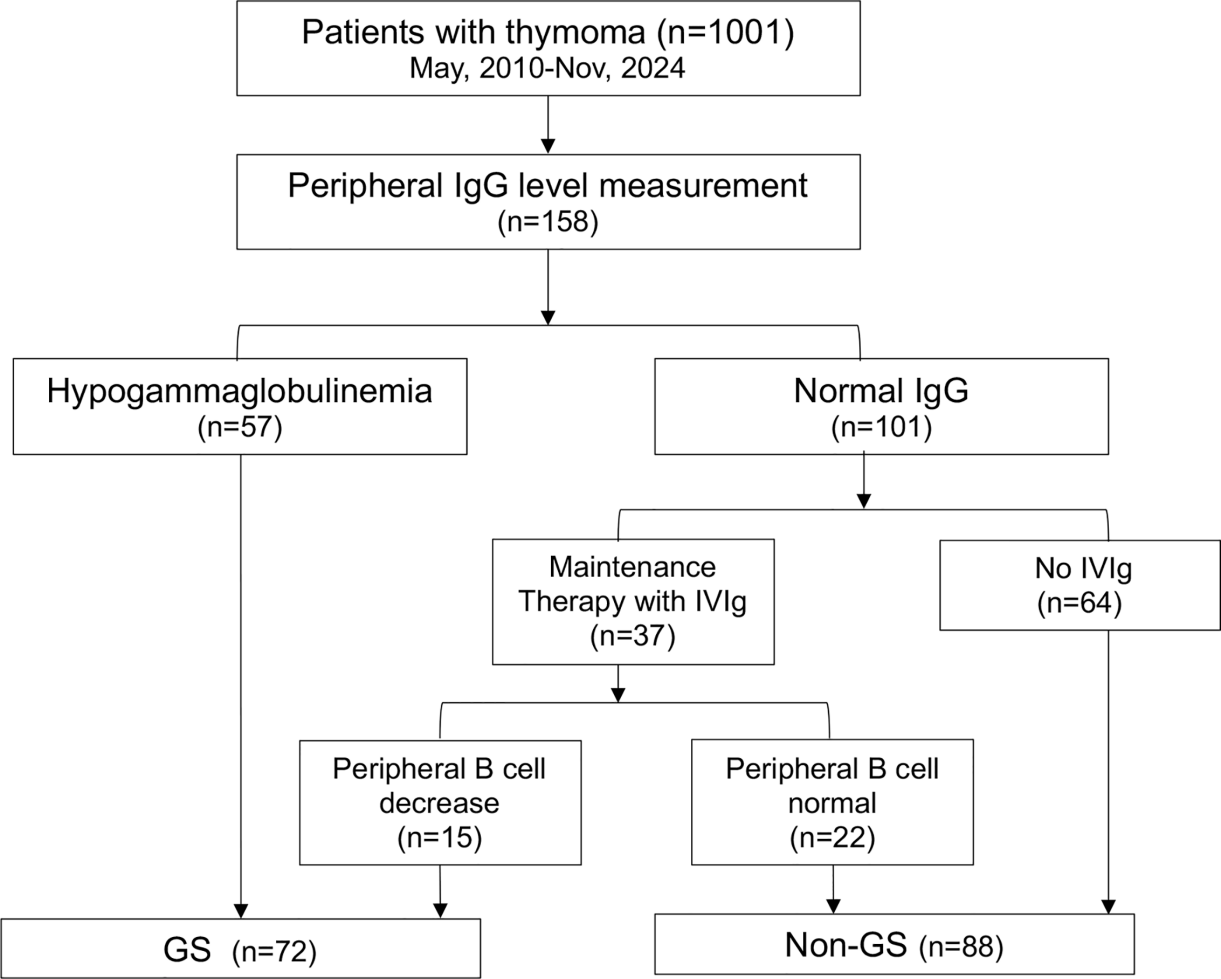
Study cohort and group definitions. A total of 158 patients with thymoma and complete immunoglobulin and lymphocyte subset data were included. Patients were classified into the Good syndrome group (n = 772) and the non-Good syndrome group (n = 888). Clinical characteristics, infection status, and survival outcomes were compared between the two groups.

Finally, we categorized the patients into two subgroups, including 72 patients with GS and 86 with non-GS (**Figure 1**). The mortality rate was higher in the GS group (15.28%, 11/72) than that in the non-GS group (8.14%, 7/86). There was no significant difference between groups in the age at evaluations for GS (53.43 ± 13.79 versus 51.21 ± 13.48 years, *P* = 0.3089), age at the diagnosis of thymoma (48.35 ± 15.43 versus 46.50 ± 14.14 years, *P* = 0.4339), the concurrence of PNS or the proportion of R0 resection for thymoma. The infection events were more frequent in the GS group in comparison to those in the non-GS group (50% versus 32.72%, *P* = 0.0384) (**Table 1**).

**Table 1:** Demographics, PNS and infections in patients with GS versus non-GS.

Variables		GS (n=72)	Non-GS (n=86)	t	*P*
% of patients		45.57%	54.43%		
Age of evaluations (y)		53.43 ± 13.79	51.21 ± 13.48	1.021	0.3089
Age at diagnosis of thymoma (y)		48.35 ± 15.43	46.50 ± 14.14	0.7846	0.4339
PNS	sPNS	56 (77.78%)	62 (72.09%)	0.5756	**0.5649**
	mPNS	16 (22.22%)	22 (25.58%)		
R0		39 (54.17%)	43 (50.00%)	χ2 = 0.2725	0.6016
Mortality		11 (15.28%)	7 (8.14%)		0.2099
Overall survival (m)		59.97 ± 73.22	65.13 ± 58.84	0.4590	0.6469
Infection		36 (50.00%)	29 (32.72%)	χ2 = 4.289	0.0384*

Comparative analysis of thymoma patients with versus without Good syndrome was performed regarding demographic characteristics, presence of paraneoplastic syndromes, mortality, survival, and infection status. The results are as follows: the mortality rate was higher in the GS group (15.28%, 11/72) than that in the non-GS group (8.14%, 7/86). There was no significant difference between groups in the age at evaluations for GS (53.43 ± 13.79 versus 51.21 ± 13.48 years, *P* = 0.3089), age at the diagnosis of thymoma (48.35 ± 15.43 versus 46.50 ± 14.14 years, *P* = 0.4339), the concurrence of PNS or the proportion of R0 resection for thymoma. The infection events were more frequent in the GS group in comparison to those in the non-GS group (50% versus 32.72%, *P* = 0.0384).

A total of 121 pathogens were identified, including bacteria (52.07%, 63/121), fungi (34.71%, 42/121) and viruses (13.22%, 16/121). Most pathogens were isolated from the respiratory tract (55.37%, 67/121), followed by urinary tract (9.09%, 11/121), bloodstream (4.13%, 5/121), gastrointestinal tract (3.31%, 4/121), and cerebrospinal fluid (2.48%, 3/121). The infection-associated mortality accounted for 44.44% (8/18) of the mortality.

**Figure 2:**
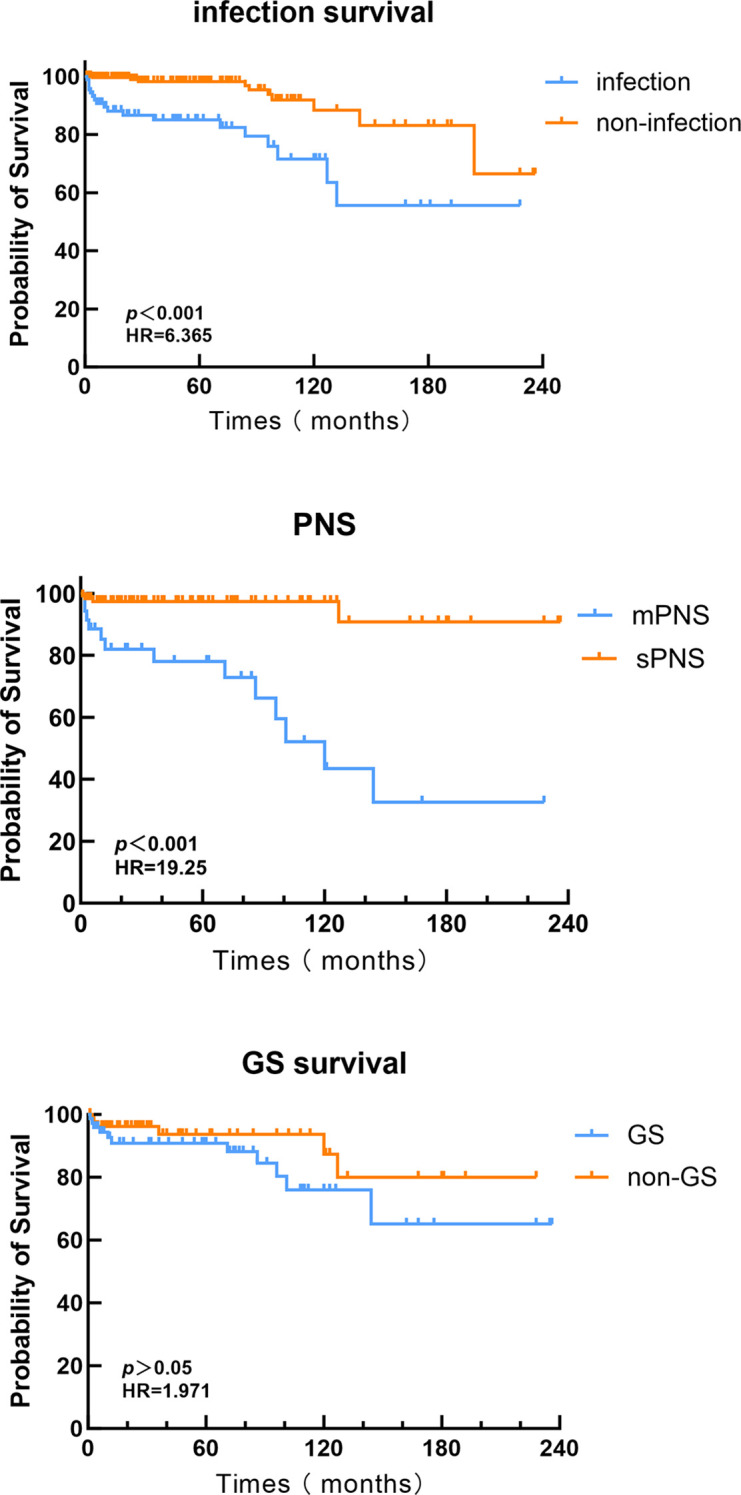
Survival outcomes in thymoma patients stratified by concurrent infections, paraneoplastic syndromes (PNS), and Good syndrome. Patients with concurrent infection showed a significantly higher risk of death (HR = 6.365, *p* < 0.05) and a reduced 10-year survival rate compared to those without infection. Patients with concomitant PNS had significantly worse 5-year and 10-year survival rates than those with isolated thymoma (both *p* < 0.001), with survival rates dropping below 80% and 50%, respectively. A poorer prognosis was also observed in patients with multiple PNS compared to those with a single PNS. Although patients with Good syndrome had a 10-year survival rate of <80% and an elevated cumulative risk of death (HR = 1.971), the difference was not statistically significant.

PNS was identified in most patients, among whom MG was the most prevalent (98.08%). Other subtypes of PNS, such as myocarditis (8.33%) and myositis (4.49%), constituted a smaller fraction of cases. Long-term survival analyses demonstrated significantly worse 20-year survival in patients with infections compared to those without infections (HR 6.365, *P* < 0.001) and in patients with multiple PNS (mPNS) compared to those with single PNS (sPNS) (HR 19.25, *P* < 0.001). However, no significant difference in 20-year survival was observed between the GS and non-GS groups (**Figure 2**).

In addition, Cox regression analysis revealed that the survival of thymoma patients was not associated with the presence of Good syndrome, gender, age, Masaoka stage, WHO classification, or R0 resection (*P*>0.05). Instead, it was correlated with early administration of intravenous immunoglobulin (IVIG) therapy (HR 0.251, 0.069-0.910), the presence of paraneoplastic syndromes (HR 11.922, 2.935-48.416), and co-existing infections (HR 2.314, 1.064-5.030) (*P*<0.05). That is, early IVIG administration reduced mortality in patients with thymoma by 74.9%, while the presence of paraneoplastic syndromes or co-existing infections increased mortality by 11.922-fold and 2.314-fold, respectively.

## Discussion

GS, a rare secondary immunodeficiency characterized by the coexistence of hypogammaglobulinemia and thymoma, was first described nearly 70 years ago (12). GS is widely regarded as a form of thymoma-associated combined immunodeficiency, involving abnormalities in both cellular and humoral immune compartments. Several reports have been published on thymoma-associated autoimmune diseases. However, studies specifically focusing on thymoma with GS remain scarce. Utilizing the International Thymic Malignancy Interest Group (ITMIG) database, Padda et al. found that MG was the most common paraneoplastic syndrome, accounting for 34% of thymoma cases. In contrast, the proportions of pure red cell aplasia (PRCA), hypogammaglobulinemia, and other immunodeficiency disorders (IDs) were less than 1% (13). Consistently, a review by Marx et al. reported an incidence of MG ranging from 30% to 44%, whereas the incidence of hypogammaglobulinemia ranged from 5% to 20% (14). Data from the Japanese Association for Research on the Thymus (JART) database further indicated that thymoma with hypogammaglobulinemia accounted for only 0.5% of all thymoma cases (15). Given the rarity of PRCA and hypogammaglobulinemia, Padda et al. noted that they were unable to independently assess the clinical impact of these syndromes (13).

Although its precise pathogenesis remains incompletely understood, accumulating evidence indicates that GS is characterized by profound immune dysregulation, including impaired T-cell development and function, marked B-cell depletion, and defective antibody production. Consequently, affected patients are exposed to a risk of recurrent or severe infections and autoimmune or paraneoplastic manifestations. Studies revealed that elevated levels of IL-1ra, IL-2, IL-4, IL-7, eotaxin, basic fibroblast growth factor (bFGF), granulocyte colony-stimulating factor(G-CSF), interferon gamma (IFN-γ), platelet-derived growth factor (PDGF), and regulated on activation, normal T cell expressed and secreted (RANTES) were significantly associated with improved overall survival (OS) in patients with TETs. Notably, after adjusting for key confounding factors including age, performance status, histological type, tumor stage, and the presence of autoimmune diseases, high levels of IL-1ra, IL-4, IL-7, PDGF, and eotaxin remained independent predictors of favorable OS (16). GS is associated with reduced life expectancy, yet large-scale long-term cohort studies remain scarce (17). In this context, this retrospective study analyses the peripheral immune profiles, infections, and PNS in patients with thymoma to explore their associations with long-term survival.

The pathogenesis of GS appears to be complex and multifactorial. Prior studies have demonstrated a marked reduction of B cells in thymoma tissues from patients with GS. This phenomenon has been attributed to inhibited B cell differentiation mediated by IL-21 signaling, and increased apoptosis driven by aberrant FasL expression, ultimately resulting in low peripheral CD19+ B cells (18). This humoral immune deficiency has been linked to increased susceptibility to opportunistic infections, such as Epstein-Barr virus (EBV) and cytomegalovirus (19, 20), which is consistent with higher infection burden observed in GS patients. Furthermore, elevated serum levels of IL-15, vascular endothelial growth factor), interferon-γ inducible protein 10, granulocyte-macrophage colony-stimulating factor, IL-6, and macrophage inflammatory protein-1α have been observed in patients with thymic tumors accompanied by autoimmune diseases (21).

The co-existence of poly-autoimmunity (e.g., single or multiple PNS) with GS is recognized but remains poorly understood. Under physiological conditions, thymic B cells, derived from early thymic precursors, exhibit high expression of MHC-II and co-stimulatory molecules, enabling effective antigen presentation and supporting CD4+ T cells interactions that contribute to Treg generation and immune tolerance (22, 23). In thymoma, cortical architectural distortion and depletion of functional thymic B cells may impair antigen presentation and Treg development, leading to defective negative selection and the peripheral escape of autoreactive T cells. This immune imbalance is consistent with the inverted CD4+/CD8+ T-cell ratios observed in patients with GS.

An important clinical observation was that in several severe cases, peripheral B-cell counts were markedly reduced despite apparently normal serum IgG levels. This discrepancy likely reflects real-world clinical practice, as these patients had received IVIg before admission or immunological workup, which may transiently normalize serum IgG levels without correcting the underlying cellular immune defect. To avoid underestimating clinically relevant immunodeficiency in such cases, reduced B-cell counts were considered supportive evidence of GS in patients with documented recent IVIg exposure. Besides, the high rate of respiratory infections was attributable to the frequent need for invasive mechanical ventilation in critically ill patients with underlying immunodeficiency.

Interestingly, our data suggest that poor long-term survival in patients with thymoma is more strongly linked to infections and mPNS than to GS alone, highlighting the critical impact of immune dysfunction and infectious complications on the clinical outcome. It is important to note, given the retrospective design of this study, that these associations do not establish causality. The observed associations between infections and survival outcomes may be confounded by factors such as overall disease severity, critical illness, and coexisting comorbidities, all of which significantly influence both infection risk and mortality.

Early identification of patients at high risk for immune-related complications is therefore crucial. Our cohort had a lower mortality (15.8%) than that previously reported (44.5%) (24), which is likely attributable to increased clinical awareness, earlier recognition of immune dysfunction, and timely initiation of IVIg and targeted anti-infective therapies. As awareness of GS and PNS continues to improve among multi-disciplinary specialists, individualized immune monitoring and infection prevention strategies may further optimize long-term outcomes.

The strength of this study include longitudinal follow-up and comprehensive analysis of immune profiles, infections, and PNS in relation to survival among patients with thymoma. However, several limitations must be acknowledged. Owing to the extended retrospective timeframe, comprehensive immunological data were only available in a subset of patients, which may introduce potential selection bias. Furthermore, the cohort consisted predominantly of patients with PNS, who often presented with poly-autoimmunity and had a history of immunosuppressant use, factors that could confound the interpretation of peripheral immune profiles. Additionally, the duration of follow-up varied among patients, with some followed for extended periods. This heterogeneity may be a source of bias and cannot be excluded as a confounding factor in survival analyses. We also acknowledge that other potential confounders, such as immunosuppressive regimens, cumulative IVIG dose, and comorbidities, could not be adequately accounted for in the analysis due to incomplete or heterogeneous documentation. Finally, the limited sample size and number of outcome events precluded robust multivariable adjustment. Therefore, the observed associations with survival should be considered exploratory rather than causal. Prospective studies with standardized immunological assessments are warranted to further clarify the complex interactions between thymoma, immune dysfunction, infection, and long-term prognosis.

## Conclusion

A substantial number of patients with thymoma exhibited GS and infections, frequently in conjunction with PNS. While GS alone was not associated with long-term survival, the presence of infections and multiple PNS was associated with reduced 20-year survival. These findings underscore the clinical relevance of immune dysfunction and infectious burden in thymoma and support the need for careful immune evaluation, timely intervention and longitudinal monitoring in this patient population.

## Data Availability

The raw data supporting the conclusions of this article will be made available by the authors, without undue reservation.
